# Fabrication of a Delaying Biodegradable Magnesium Alloy-Based Esophageal Stent via Coating Elastic Polymer

**DOI:** 10.3390/ma9050384

**Published:** 2016-05-17

**Authors:** Tianwen Yuan, Jia Yu, Jun Cao, Fei Gao, Yueqi Zhu, Yingsheng Cheng, Wenguo Cui

**Affiliations:** 1 Department of Radiology, Shanghai Jiao Tong University Affiliated Sixth People’s Hospital, Shanghai Jiao Tong University, 600 Yi Shan Road, Shanghai 200233, China; ytw806@163.com; 2 Department of Orthopedics, The First Affiliated Hospital of Soochow University, Orthopedic Institute, Soochow University, 708 Renmin Road, Suzhou 215006, China; jiayu@suda.edu.cn; 3 Department of Interventional Oncology, Dahua Hospital, Xuhui District, Shanghai 200237, China; 13611890000@163.com; 4 Zhejiang Zylox Medical Device Co., Ltd., No. 1500, Wenyi West Road, Hangzhou 311121, China; fee_go@163.com

**Keywords:** benign esophageal stricture, biodegradable stent, magnesium, PCL-PTMC membrane

## Abstract

Esophageal stent implantation can relieve esophageal stenosis and obstructions in benign esophageal strictures, and magnesium alloy stents are a good candidate because of biodegradation and biological safety. However, biodegradable esophageal stents show a poor corrosion resistance and a quick loss of mechanical support *in vivo*. In this study, we chose the elastic and biodegradable mixed polymer of Poly(ε-caprolactone) (PCL) and poly(trimethylene carbonate) (PTMC) as the coated membrane on magnesium alloy stents for fabricating a fully biodegradable esophageal stent, which showed an ability to delay the degradation time and maintain mechanical performance in the long term. After 48 repeated compressions, the mechanical testing demonstrated that the PCL-PTMC-coated magnesium stents possess good flexibility and elasticity, and could provide enough support against lesion compression when used *in vivo*. According to the *in vitro* degradation evaluation, the PCL-PTMC membrane coated on magnesium was a good material combination for biodegradable stents. During the *in vivo* evaluation, the proliferation of the smooth muscle cells showed no signs of cell toxicity. Histological examination revealed the inflammation scores at four weeks in the magnesium-(PCL-PTMC) stent group were similar to those in the control group (*p* > 0.05). The α-smooth muscle actin layer in the media was thinner in the magnesium-(PCL-PTMC) stent group than in the control group (*p* < 0.05). Both the epithelial and smooth muscle cell layers were significantly thinner in the magnesium-(PCL-PTMC) stent group than in the control group. The stent insertion was feasible and provided reliable support for at least four weeks, without causing severe injury or collagen deposition. Thus, this stent provides a new stent for the treatment of benign esophageal stricture and a novel research path in the development of temporary stents in other cases of benign stricture.

## 1. Introduction

Diseases in the esophageal atrium often result in esophageal benign strictures. Also, this type of stricture can be caused by external pressure on the mucosal ring and the mediastinal tissue. A benign stricture of the esophagus (BSE) can be responsible for complications such as aspiration, weight loss, and malnutrition, thus reducing a patient’s quality of life [1]. To combat these side effects, the use of an esophageal stent implantation has been used on patients with dysphagia with excellent results. Furthermore, clinical research [2,3,4] supports this surgical method.

The materials mainly used in stents for BSE are either metal or polymer. The advantages of a metal stent are its high strength and structural stability. Song *et al.* first designed a stainless steel-coated stent, and then later this design was replaced with a nickel titanium alloy stent. These stents produced positive results [5,6,7,8]. However, due to the strength and stiffness of the metal, it can be difficult to remove. Consequently, removal of the stent can lead to pain, bleeding, perforation, and other complications [9].

Therefore, the ideal stent should be a completely biodegradable orthopedic stent. After being implanted, a stent can act as a mechanical support over a short period of time. When the therapeutic process is completed, the stent will degrade fully to avoid further foreign body stimulation to the esophageal tissue and prevent restenosis. A newly developed biodegradable implant material made out of a magnesium alloy has outstanding biocompatibility and mechanical properties. The implant is attracting the attention of a growing number of researchers. Magnesium-based alloys have a high specific strength, specific stiffness and good processability. After being treated by a special technique, the magnesium alloy has enhanced anti-corrosive properties and mechanical properties, as well as a longer degradation time [10]. However, the original corrosion resistance property of the magnesium alloy is poor, especially in a corrosive environment containing chloride ions. The esophageal environment, which is typically a corrosive environment, has strict requirements for stent materials. Hence, in terms of the characteristics of magnesium alloys, it has important potential applications in clinical practice to develop biodegradable esophageal stents where corrosion is minimal.

**Figure 1:**
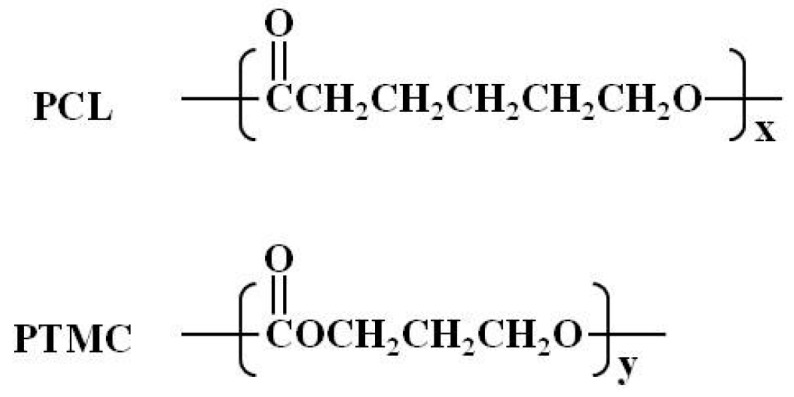
Chemical structures of PCL and PTMC.

Alternatively, polymer materials are best when good flexibility and high tensile strength are desired properties. The treatment of an esophageal stricture, esophageal fistula, and postoperative anastomotic fistula is prevalent in literature [11,12,13,14,15]. Combining the two previously mentioned types of treatments, currently existing stents consist of a polymer membrane coating on a metal stent. This method maintains the mechanical performance while improving the implantation and removal process. Two polymers commonly used are Poly(ε-caprolactone) (PCL) and poly(trimethylene carbonate) (PTMC) (Figure 1). These aliphatic polyesters are biodegradable and biocompatible. They degrade at different rates and are used in different biomedical applications. With uses in creating scaffolds for tissue engineering, PCL is a non-immunogenic semi-crystalline polymer with slow biodegradability and good drug permeability [16]. On the other hand, PTMC is an amorphous biopolymer with good mechanical resistance and high thermal and chemical stability. It is elastic at ambient temperature. PTMC was shown to not have any influence on heart, liver, and kidney tissues through *in vivo* biocompatibility and toxicity assays [17]. Furthermore, combining these two polymers into the PCL-PTMC mixed polymer has shown potential for surgery and nerve guide repairs. It is highly biocompatible and has controllable mechanical properties and degradation rates [18,19]. The use of this mixing polymer as a coating membrane has not yet been reported.

This experiment fabricates completely biodegradable esophageal stents by coating an elastic and biodegradable PCL-PTMC mixed polymer film on the degradable magnesium-based stent surface. The stent is completely degradable and has similar mechanical properties to metal. In addition, a large molecular weight polymer surface coating can prevent the magnesium alloy from being drastically corroded by the corrosive environment where it is implanted. The purpose of this research also includes investigating the biomechanical properties, assessing the safety of the stent for cells *in vitro* and assessing extensibility and safety performance *in vivo*.

## 2. Materials and Methods

### 2.1. Materials

The magnesium-(PCL-PTMC) stent has two components: a magnesium stent coated with a PCL-PTMC membrane. Using a commercial magnesium alloy (Sanming, Biomedical Company, Yangzhou, China), the bare stent was constructed with 0.20 mm magnesium alloy (AZ31, Mg-3Al-1Zn). AZ31 has the following chemical composition (in mass %): 3% Al, 1% Zn, and 0.43% Mn and Mg (balance).

Minghe Functional Polymer Co., Ltd. (Qingdao, China) supplied the PCL and PTMC. The PCL-PTMC membrane coated the stent with the dipping and spinning method (Figure 1). The magnesium wire framework was built using precise aluminum molds of the stent. A 1:1 ratio of PCL to PTMC was blended then dissolved in the solvent dichloromethane. The magnesium stent was then dipped into the polymer mixture five times and cured for 6 h at 40 °C. The molds were cooled for 3 h in ambient conditions and the stents were removed. By this process, the membrane’s shape will remain constant after its removal from the stent. A mark was placed at the distal end to ensure accurate and precise positioning under fluoroscopy.

### 2.2. Magnesium-(PCL-PTMC) Stents Description

The magnesium alloy wires were formed to create a cylindrical, cross-linked mesh stent with a 14 mm cydariform shape at the head and a tubiform shape at the distal end to prevent movement of the stent. The entire magnesium stent was coated in the PCL-PTMC membrane. The dimensions, diameter by length, are 10 mm by 31 mm when fully expanded. A trisected anti-reflux valve was implemented where the body meets the tail. A 6-mm-wide (approximately 18 French) delivery system was used to compress and insert the stent. The entire stent was radiopaqued so that it could be positioned accurately under the fluoroscope.

### 2.3. Stent Radial Force Test

The resist extrusion performance and the force supported in the radial direction were determined by measuring the compression recovery characteristics and the radial forces of the stent at a dry station. The stent compression was measured between a pressure head (5 mm/in) width, with a 0.1 mm/s (downside) and a flat plate using the Instron 5272 Advanced Materials Testing System (Instron Corporation, Norwood, MA, USA). The stability of the stent against outer radial forces was represented by the radial force curves. This test was repeated 48 times.

### 2.4. In Vitro Degradation Evaluation

Mass lost from the stent was used to quantify the degradation of the stent magnesium and the PCL-PTMC membrane. This was evaluated by using 1 × 1 mm^2^ sections of the stents. These pieces were weighed and then incubated at 37 °C in 20.0 mL of two phosphate-buffered saline (PBS) solutions with pH values of 7.4 and 4.0. At several time points in the process, triplicate stent samples were recovered. These were then rinsed with distilled water and dried to a constant weight in vacuum desiccators. Mass loss was obtained by comparing the mass at that point in time to the original dry weight.

### 2.5. Stent Insertion

All protocols were approved by the animal research committee of the Orthopedic Institute of Soochow University and were conducted in accordance with the guidelines of the International Council on Animal Care (US National Institutes of Health and European Commission). The subject group consisted of 10 healthy New Zealand rabbits that were between the ages of five and eight months old. Both sexes of rabbit were tested and the weight varied from 2.3 to 3.8 kg. The rabbits were randomly divided into a stent group and a control group (*n* = 5 each). The stent was inserted into the lower third of the esophagus of the stent group rabbits using fluoroscopic guidance. The control group did not receive stents.

The magnesium-(PCL-PTMC) stents were placed in the lower third of the esophagus of the rabbits in the stent group. A stiff exchange wire (0.035 in × 260 cm, diameter × length, Terumo, Tokyo, Japan) was threaded through the mouth and into the stomach under fluoroscopic guidance. This wire was used as a guide to deliver the stent into the lower third of the esophagus. Using fluoroscopic guidance and esophagographic images, the stent was released. To achieve full expansion, a balloon catheter (10 mm × 40 mm) was inflated within the stent. Further esophagography was done to verify the extent of stent expansion and esophageal perforation. Rabbits from the control group did not receive a stent.

#### Pneumatic Dilation Procedure

The 75-cm-in-length (Changhong Medical Instrument Co., Ltd., Changzhou, China) balloon catheters (10 mm × 40 mm) were used in this study. All the rabbits were fixed in the supine position. After a 260-cm-long stiff exchange wire (Terumo, Tokyo, Japan) was passed through the esophageal and into the gastric cavity, the balloon catheter was advanced over the guide wire and positioned across the diaphragmatic hiatus using radiopaque markers as a guide. The balloon was then inflated for 10 to 20 s until the stents was expanded. After the stent’s dilation a transcatheter gastrograffin injection was performed right now to prevent the occurrence of esophageal perforation.

### 2.6. Follow-Up

At four weeks after the stent insertion, an esophagography was performed. This was done in the upright position under general anesthesia. These three sets of esophagography were compared for stent migration, patency, and the diameter of the esophagus with the treatment.

### 2.7. Histological Examination

Both groups of samples were submerged in 10% neutral-buffered formalin for at least 48 h. They were then put through a gradient of 70% to 100% ethanol solutions and embedded in paraffin. Several cross-sections were stained with hematoxylin and eosin (HE) to evaluate the inflammatory response on revised inflammation scores [20]. Submucosal collagen deposition was assessed using Masson trichome staining. Mouse anti-proliferating cell nuclear antigen (PCNA) antibody (1:100 dilution; NeoMarkers, Thermo Fisher Scientific Inc., Fremont, CA, USA) and mouse monoclonal α-smooth muscle actin (α-SMA) antibody (1:50 dilution; Santa Cruz Biotechnology Inc., Dallas, TX, USA) were used to immunostain the esophageal samples using the Elivision immunohistochemical technique. Negative controls omitted the primary antibodies. The pathologist responsible for the review and analysis of specimens in this experiment was blinded and unaware of the animal randomization, treatment procedure, and follow-up protocols. 

### 2.8. Statistical Analysis

Statistical analysis was performed using GraphPad Prism 5.0 software (GraphPad Software Inc., San Diego, CA, USA). Nonparametric data was compared with the Fisher exact test. Categorical values were expressed using numbers or percentages and continuous numerical variable were shown as a mean ± standard deviation. The overall change in esophageal diameter at each point in time was compared using a one-way and two-way ANOVA (analysis of variance). This analysis was also used to compare the PCNA proliferation index and the collagen area at each time point within or between the two sample groups. Prior to one-way ANOVA, the Shapiro-Wilk test was used to assess the homogeneity of variance and normal distribution of the dependent variables. Results that are *p* < 0.05 were defined to be statistically significant.

## 3. Results

### 3.1. Macro Structure and Clinical Usability

**Figure 2:**
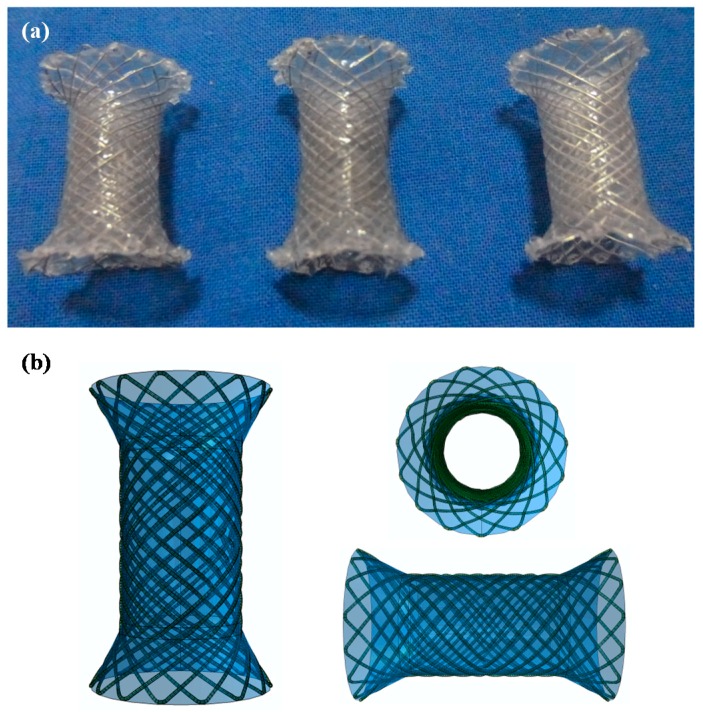
(**a**) Photographs of the perfecting opened PCL-PTMC coated magnesium-stent shape; (**b**) the covered membrane stent’s different profile diagrammatic cross-section.

Figure 2 depicts the opened bare stents and related parameters. The stent consists of a cross-linked magnesium alloy wire cylindrical mesh body. The stent is cylindrical in shape with a 15 mm cydariform at the head and a 5 mm tubiform at the distal end to prevent migration at the gastroesophageal junction. The body of the stent and the distal end are coated with the PCL-PTMC membrane. The diameter varies from 10 mm at the body and 15 mm at the head and distal end. When fully expanded, the stent length is 25 mm. There is a trisected anti-reflux valve where the stent body meets the tail.

### 3.2. Mg-PCL-PTMC Stent and Its Mechanical Evaluation

**Figure 3:**
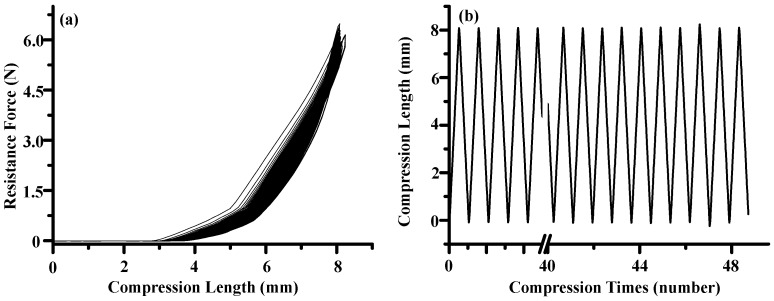
(**a**) Mechanical compression curve analysis of the stent; (**b**) compression-recovery curves of length and time in repeated compression tests. (*n* = 5, constant pressure = 10 N).

The tensile stress and strain for the magnesium (PCL-PTMC) stents were tested to determine the mechanical properties shown in Figure 3a,b. The PCL-PTMC membrane had a uniform thickness of 100 μm, and was tightly wrapped and fixed to the cross-linked, knitted, bare magnesium mesh tube. The tube was able to maintain its size and morphology due to its excellent flexibility and elasticity. The magnesium-PCL-PTMC stent displayed good elastic deformation properties with no tearing or ablations. The stent was flattened and modified enough to lose elasticity after 48 repeated compressions (compression distance: 0–8 mm). The curves shown in Figure 2a show a radial force of 0.65 ± 0.23, 1.79 ± 0.17, and 5.91 ± 0.25 N when compressed at 4, 6 and 8 mm, respectively. Figure 3a reveals that at the same compression rate of each stent, a stronger compression load and a lower spring-back displacement are needed for the stent. Thus, the PCL-PTMC-coated magnesium stents possess good flexibility and elasticity, and could provide enough support against lesion compression when used *in vivo*.

### 3.3. In Vitro Biodegradation of Mg-(PCL-PTMC) Stent

**Figure 4:**
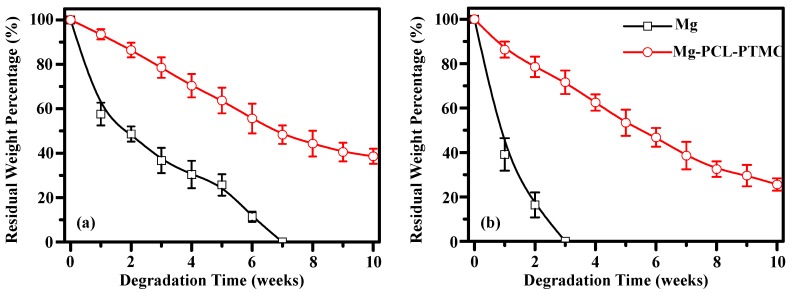
Analysis of the degradation of the bare magnesium and magnesium-(PCL-PTMC) stent samples in phosphate-buffered saline with pH values of 7.4 (**a**) and 4.0 (**b**).

The degradation behavior of the magnesium and magnesium-(PCL-PTMC) stents were determined in terms of the amount of mass loss in buffer solutions of pH 7.4 and 4.0 (Figure 4). In neutral PBS (pH 7.4) the bare magnesium wires degraded at a faster rate than the (PCL-PTMC)-coated magnesium wires. The residual mass of the bare magnesium wires was 57.3% ± 3.6%, 42.5% ± 4.2% and 30.2% ± 3.8% compared to 95.3% ± 2.3%, 90.5% ± 2.6% and 82.6% ± 3.4%, respectively, for the (PCL-PTMC)-coated wires, at one, two and four weeks, respectively (*p* < 0.01). Furthermore, the (PCL-PTMC)-coated stents showed only 40% mass loss at 10 weeks, indicating that the PCL-PTMC membrane significantly slowed stent degradation in neutral PBS (pH 7.4). The degradation rate of the bare magnesium wires was faster in acidic PBS (pH 4.0) than in neutral PBS (pH 7.4); at two weeks, over 80% mass was lost in the acidic solution. In comparison, the PCL-PTMC membrane-coated wires showed greatly reduced degradation in acidic PBS at one (95.5% ± 2.6% *vs.* 32.3% ± 7.4%), two (89.5% ± 5.2% *vs.* 15.4% ± 4.1%) and four (72.5% ± 4.8% *vs.* 0) weeks (*p* < 0.01; Figure 4). Therefore, PCL-PTMC membrane-coated magnesium was a good material combination for the fabrication of biodegradable stents in terms of possible retention time.

### 3.4. Intervention Procedure

**Figure 5:**
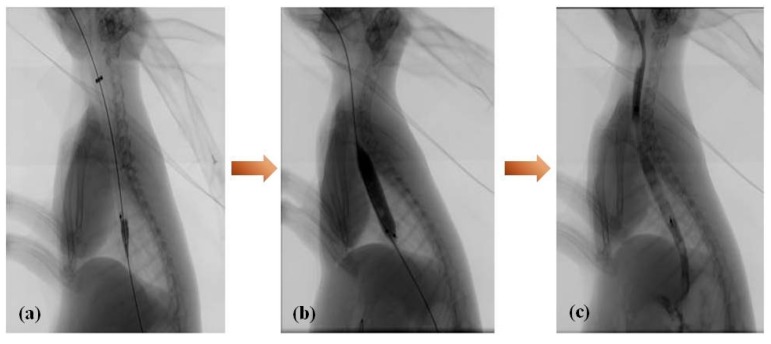
Esophagography during stent insertion (**a**); balloon dilation after magnesium-(PCL-PTMC) stent insertion (**b**) and immediate esophagogram (**c**) reveals that the stented esophagus is well patent.

Stenting was successful in all 10 rabbits in the magnesium-(PCL-PTMC) stent group. All rabbits tolerated the procedure well and procedure-related adverse events, including esophageal perforation and bleeding, did not occur during or following stent insertion. Esophagography revealed that the contrast agent passed smoothly through the stented esophagus in both groups (Figure 5). No immediate stent migration into the stomach occurred after stent placement in the magnesium-(PCL-PTMC) stent group.

### 3.5. Follow-Up

Out of all the rabbits in both groups that underwent regular esophagography, none have died during the follow-up. Two rabbits from the magnesium-(PCL-PTMC) group had the stent migrate from the original location. The esophageal diameter in the magnesium-(PCL-PTMC) stent group was 9.09 ± 0.85 mm after stent insertion, which was larger than the corresponding values in the control group (8.35 ± 0.83 mm; *p* < 0.05). Follow-up esophagography revealed no in-stent stenosis in the magnesium-(PCL-PTMC) stent group. In this group, the mean diameter of the stented esophagus (8.74 ± 0.81 mm) at the end of follow-up (four weeks) was similar to that observed immediately after stent insertion (9.09 ± 0.85 mm, *p* > 0.05) and that in the control group at four weeks (8.68 ± 0.76, *p* > 0.05).

### 3.6. Histological Study

**Figure 6:**
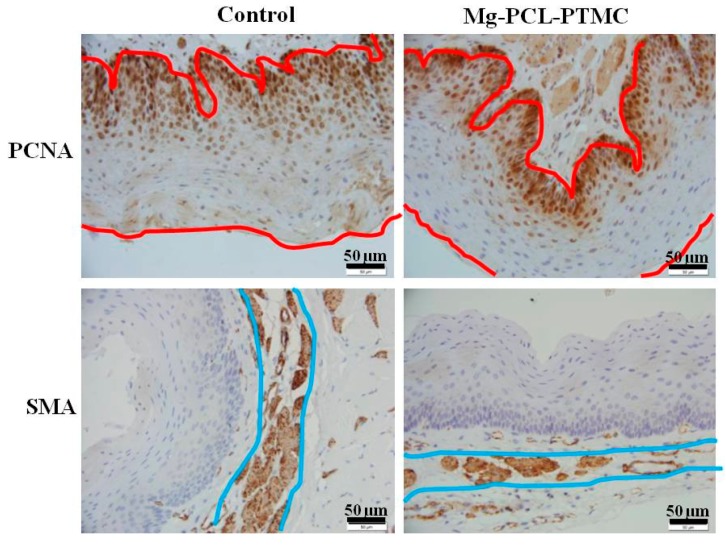
Both the epithelial and smooth muscle cell (SMC) layers were significantly thinner in the magnesium-(PCL-PTMC) stent group than in the control group (magnification ×400, the red and blue lines indicate the thickness of the epithelial and SMA layers, respectively).

**Figure 7:**
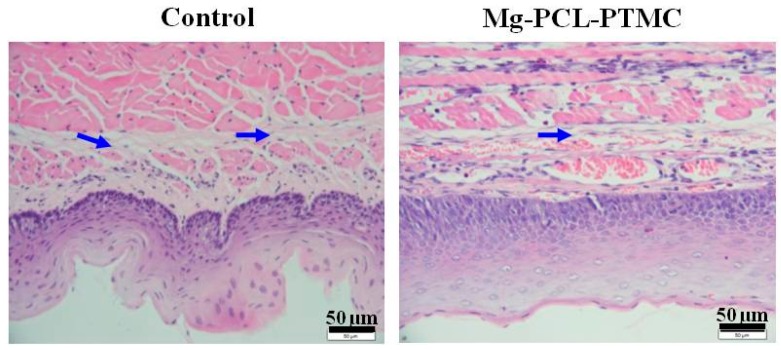
HE trichrome staining shows collagen deposition in the submucosa in the control and magnesium-(PCL-PTMC) stent groups. *p* < 0.05 for comparisons between the control and magnesium-(PCL-PTMC) stent groups.

HE staining revealed significantly less esophageal wall remodeling in the magnesium-(PCL-PTMC) stent group than in the control group. The inflammation scores at four weeks in the magnesium-(PCL-PTMC) stent group were similar to those in the control group (*p* > 0.05). PCNA-positive cells (squamous epithelial cells) were mainly found in the epithelial layer; squamous epithelial cells located near the lumen and close to the stent were mostly negative for PCNA. The epithelial layer, as shown on PCNA staining, was much thinner in the stent group than in the control group. Immunostaining revealed that the SMA layer in the media was thinner in the magnesium-(PCL-PTMC) stent group than in the control group (*p* < 0.05). Masson staining revealed that collagen was mainly localized in the submucosa, similar between the stent and the control groups at each follow-up time point, indicating that the tissue reaction followed by stent dilation injury and degradation was similar to that in the control (Figure 6 and Figure 7).

## 4. Discussion

Recently, researchers have come up with the concept of a biodegradable stent, which is defined as a stent that provides mechanical support for a designated period and then self-degrades gradually until it completely disappears. The mechanical stimulation to target the esophagus and resulting adverse reactions, such as partial chronic inflammation and intima and smooth muscle hyperplasia, do not exist after the stent is fully degraded. In terms of different materials, two types can classify current clinical degradable stents: (1) a biodegradable metal stent; (2) a biodegradable high polymer stent [21,22,23,24,25,26,27]. Presently, there is no ideal stent that can successfully cure esophageal benign stricture as well as reduce the existence of complications. Fully considering restenosis and the difficulty in removing a metal stent, hard implantation and inadequate bearing force, we designed a new degradable esophageal stent by coating a biodegradable magnesium alloy with a degradable aliphatic polyester (PCL-PTMC). This stent has outstanding degradability, elasticity, flexibility and histocompatibility as well as a light foreign body sensation, which satisfies the clinical practice requirements.

In this experiment, the stent’s characteristics are as follows: (1) magnesium-(PCL-PTMC) stents provided reliable radial force, with the stents providing good support for long periods of time *in vivo* and the PCL-PTMC membrane greatly reduced the magnesium biodegradation rate *in vitro*; (2) the procedural success rate of magnesium-(PCL-PTMC) stent insertion was 100%; (3) stenting effectively resulted in esophageal wall remodeling and minimized injury as well as inflammatory reaction; (4) the stent provided excellent biocompatibility; (5) the coating design can be adjusted if the stent is translocated.

Mechanical tests showed that under different compression distances, this stent has excellent recovery properties. After nearly 48 compressions, the material could still maintain its original mechanical compressive strength. This illustrates that the (PCL-PTMC)-stabilized magnesium alloy tubular stent can maintain its mechanical properties and structural stability over extended use.

The stent degradation rate directly affects the structural support provided by the stent. *In vitro* experiments revealed that the PCL-PTMC coating lengthened the degradation time of the magnesium alloy. In neutral PBS, 65% of the original mass remained after 10 weeks, while in acidic PBS, complete degradation occurred after 10 weeks. All the above data indicate that magnesium-(PCL-PTMC) stents can effectively provide good support.

There are no notable complications such as esophageal perforation, bleeding and stent translocation that occurred in the experiment. The stent translocation rate is low in this experiment, but if a larger sample is used or the original sample is observed for a longer time, the translocation rate could be higher. It is confirmed by our group that the stent design has satisfactory therapeutic effect for rabbit esophageal benign stricture. The reaction to surrounding tissue and mucosa is low. It is difficult to hurt the esophagus and translocate. The stent is able to continuously and steadily expand. It is simple to operate and control. The application prospects of it are clear and broad. The new design provides a new method and theory basis for esophageal benign stricture therapy, as well as a new idea for benign stricture stent therapy in other parts of body.

Although the results from these experiments are good, some limitations exist for the stent. The magnesium-(PCL-PTMC) stent was inserted into the normal esophagus and the tissue reaction in the normal esophagus may differ from that in an esophageal stricture. This stent should be applied in a BSE model to fully investigate its feasibility, efficacy and tissue reaction *in vivo*. The duration of the *in vivo* experiments spanned a short observation time and effective support was provided for only two weeks. An extensive test should be carried out to observe the long-term effectiveness of the stent. The recurrence case in the later period of the experiment and forward complications were not observed as much. Further research is required to delay biodegradation and improve the duration of support. The efficacy, optimal implantation time and tissue reaction *in vivo* require a longer follow-up study.

## 5. Conclusions

The design of the magnesium-(PCL-PTMC) stent was able to carry out all requirements which included excellent tensile strength and good security with few complications. It is possible that this esophageal stent would be able to yield good results in the treatment of BSE based on the results of this experiment. The stent is unlikely to damage the esophagus, and it remained fixed in position while having a persistent force-expansion. The operation is simple with strong controllability, and thus the stent has high prospects for clinical application. It provides a new stent for the treatment of benign esophageal stricture and a novel research path in the development of temporary stents in other cases of benign stricture.
